# Reduction in lower urinary tract mucosal microtrauma as an effect of reducing eyelet sizes of intermittent urinary catheters

**DOI:** 10.1038/s41598-024-65879-4

**Published:** 2024-07-01

**Authors:** Andreas Willumsen, Tabasum Reza, Lars Schertiger, Per Bagi, Michael Kennelly, Lene Feldskov Nielsen

**Affiliations:** 1 Coloplast A/S, Holtedam 1, 3050 Humlebaek, Denmark; 2 Department of Urology, Rigshospitalet, Copenhagen, Denmark; 3 Department of Urology, Carolinas Medical Center, Charlotte, North Carolina USA

**Keywords:** Urology, Lower Urinary Tract, Urethra, Bladder, Intermittent urinary catheter, Mucosal suction, Pressure peaks, Urothelium trauma, Microtrauma, Micro-hole Zone Technology, Conventional eyelet catheter, And Biomedical Engineering, Urology, Biomedical engineering, Risk factors

## Abstract

Intermittent catheterization (IC) utilizing conventional eyelets catheters (CECs) for bladder drainage has long been the standard of care. However, when the tissue of the lower urinary tract comes in close proximity to the eyelets, mucosal suction often occurs, resulting in microtrauma. This study investigates the impact of replacing conventional eyelets with a drainage zone featuring multiple micro-holes, distributing pressure over a larger area. Lower pressures limit the suction of surrounding tissue into these micro-holes, significantly reducing tissue microtrauma. Using an ex vivo model replicating the intra-abdominal pressure conditions of the bladder, the intra-catheter pressure was measured during drainage. When mucosal suction occurred, intra-catheter images were recorded. Subsequently affected tissue samples were investigated histologically. The negative pressure peaks caused by mucosal suction were found to be very high for the CECs, leading to exfoliation of the bladder urothelium and breakage of the urothelial barrier. However, a micro-hole zone catheter (MHZC) with a multi-eyelet drainage zone showed significantly lower pressure peaks, with over 4 times lower peak intensity, thus inducing far less extensive microtraumas. Limiting or even eliminating mucosal suction and resulting tissue microtrauma may contribute to safer catheterizations in vivo and increased patient comfort and compliance.

## Introduction

Intermittent catheterization (IC) is a safe, easy, and effective standard of care method used 4–6 times a day for the urological management in patients with urinary incontinence^1–8^. The method reportedly improves patient quality of life and has a lower incidence of bladder stones, reduced bladder capacity and UTIs compared to other approaches^9–11^.

The devices typically used for IC are conventional eyelet catheters (CECs). In spite of many advances in their design and of their many advantages, patients using CECs sometimes report a pinching sensation towards the end of catheterization. This is thought to be caused by bladder mucosa being suctioned into the eyelets towards the end of voiding. While this phenomenon is poorly understood as it has not been investigated in vivo*, *ex vivo experiments^12^ demonstrated that flow stops during IC were linked to mucosal suction, showing tissue being sucked into the catheter when the last eyelet was occluded. The frequency of mucosal suction was reported to be between 60 – 100%, depending on the brand of CEC tested. Repositioning of the CECs did not prevent further mucosal suction.

As IC is performed multiple times per day^1,3,4^, it is reasonable to assume that patients are susceptible to multiple mucosal suction episodes daily. Such events are not only uncomfortable but may lead to areas of mucosal microtrauma and poor compliance to IC resulting from patient dissatisfaction^7,13^. Furthermore, as it has been shown that trauma related to catheter insertion and removal may contribute to UTIs^14^, it is not far-fetched to imagine that trauma caused by other processes may also be a contributing factor. While there are literature reports investigating mucosal suction and its harmful impact on the bladder tissue in animal models using indwelling catheters^15–17^, no such studies for CECs exist to date and the questions about bladder microtrauma in IC remain unanswered.

As of yet, using hydrophilic coatings on CECs has been shown to reduce the risk of urethral microtrauma^7,18,19^, but no investigations have been conducted to assess the potential role of pressure dynamics inside the catheter close to the suction site in causing microtrauma. Furthermore, while CEC chemical surface modifications have long been employed to improve safety, changes to the device eyelets and their potential benefits have not been explored to the same extent. Recent studies *ex vivo*^20^ and *in vivo*^21,22^ indicate catheters with many (around 80) micro-holes with diameters of below 1 mm – instead of the 2 large eyelets of CECs – may positively impact device performance and safety.

In this context, the current study aimed to fill the knowledge gap of understanding the impact of mucosal suction events through CECs and the extent of the resulting microtrauma. Furthermore, this work intended to test the potential of the new Micro-Hole Zone Catheters (MHZCs) mentioned above to limit the extent of microtrauma during catheterization, building on suggestions from literature that mucosal suction is limited with this new device^20^. Published studies on the MHZCs^20–22^ show that far fewer flow-stop events resulting from tissue blocking the holes and potentially being sucked through them occur compared to CECs. As such, in order to be able to study such events for the MHZCs, the current experiments did not use the full 80 + micro-holes the new devices typically have, but instead investigated scenarios with far fewer (2 or 8) micro-holes. Tests comparing the pressure profiles for CECs and MHZCs during bladder emptying were conducted and the impact of mucosal suction events on macro (naked eye) and micro (histology) levels are presented in the following.

## Results

### Endoscopic observations and visual inspection of the mucosa

**Figure 1:**
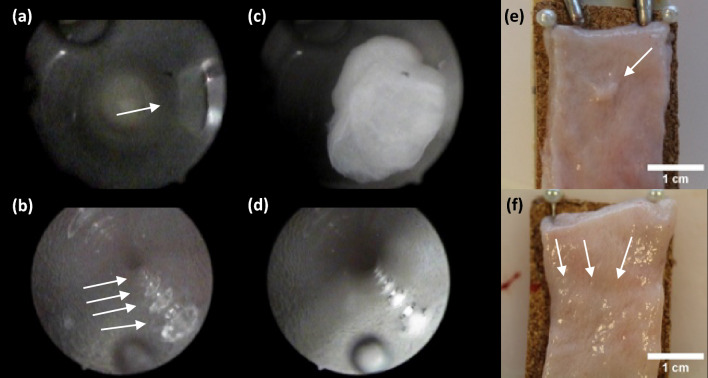
Endoscopic observations and visual inspection of the mucosa. (**a**–**d**): Intraluminal endoscopic images in CEC before and during drainage (**a** and **c**, respectively), and in MHZC before and during drainage (**b** and **d**, respectively), white arrows denote eyelet (**a**) and micro-holes (**b**). Visual inspection of mucosal suction shows distinct suction marks from CECs while the suction marks were less conspicuous and difficult to locate with the naked eye for the MHZCs.

The local hydrodynamic suction, caused by the moving column of water through the CECs, sucked the sample tissue into the catheter lumen through the eyelet. This resulted in the eyelet being blocked instantaneously, stopping the flow through the catheter. The hydrostatic suction, caused by the static water column inside the model reservoir, kept the bladder mucosa sucked into the eyelet. The suction was sufficient to keep the bladder tissue stuck inside the catheter (Fig. 1c). Removal of the bladder tissue from the eyelet revealed distinct suction marks left by all tested CECs (Fig. 1e). The mucosal suction caused by the MHZCs was far less obvious. Most of the samples had small circular marks, which were barely visible by naked eye and difficult to capture with a camera (Fig. 1f).

Compared to the unblocked eyelet in Fig. 1a, it is evident in Fig. 1c that a significant part of the mucosa is sucked into the lumen of the CECs, resulting in the suction mark presented in Fig. 1e. The MHZCs present negligible ingress of the mucosa into the catheter lumen due to the smaller area of the micro-holes compared to the CEC’s large eyelets, as seen when comparing Figs. 1b (no suction) and 1d. In MHZCs, the minor suction is limited solely to the micro-holes themselves (Fig. 1d).

### Mucosal suction pressure peaks

**Figure 2:**
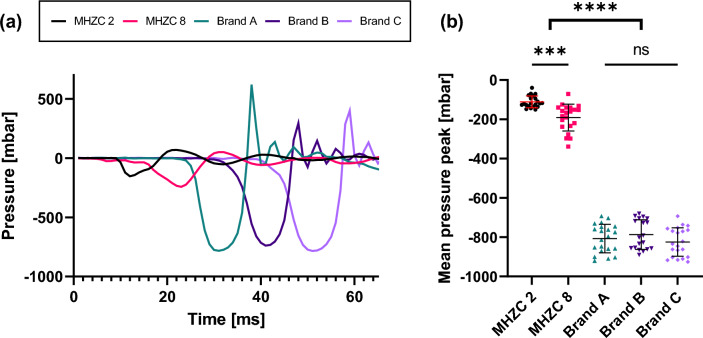
Pressure recording for the different catheters. (**a**): Staggered (10 ms) examples of traces of pressure readings during the initial phase of mucosal suction for each catheter type on the same bladder specimen. The main pressure event only lasts for 10–15 ms for all catheters. Ten of these events were performed for each run. The mean was calculated and used as a single data point in (**b**): Mean peak pressure for tissue sample by catheter. A significant difference between the prototype MHZCs vs. the CECs was found (p < 0.0001, Dunnet’s T3 multiple comparisons t-test).

Figure 2a presents representative examples of pressure data traces for a single mucosal suction event for each of the different catheter types. For Brand B, 2 runs were omitted because of faulty readings, resulting in a total of 19 runs for this catheter type. A clear difference between the CECs and MHZCs is evident with the CEC pressures reaching more extreme negative values. This is further supported in Fig. 2b where the averages of the 10 mucosal suctions were plotted as a single data point in the scatter plot. The mean negative peak pressures for the CECs were found to be significantly different from those of the MHZCs (p < 0.0001). No significant differences were found among the CECs (p values ranging from 0.6707 to 0.9944). The summary statistics for the pressure study are presented in Table 1.

**Table 1:** Summary statistics of the mean of peak pressures measured during mucosal suction for CECs and MHZCs.

Catheter	Negative pressure peak		Standard Deviation	
	Avg. (mbar)	Range (mbar)	SD (mbar)	Relative (%)
CEC Brand A	− 806.8	[− 692; − 921]	72.37	8.97%
CEC Brand B	− 795.8	[− 679; − 890]	75.28	9.57%
CEC Brand C	− 831.0	[− 692; − 926]	72.56	8.80%
MHZC 2 holes	− 110.3	[− 39; − 151]	29.82	26.84%
MHZC 8 holes	− 192.4	[− 71; − 339]	68.13	35.63%

### Histological assessment of microtrauma

**Figure 3:**
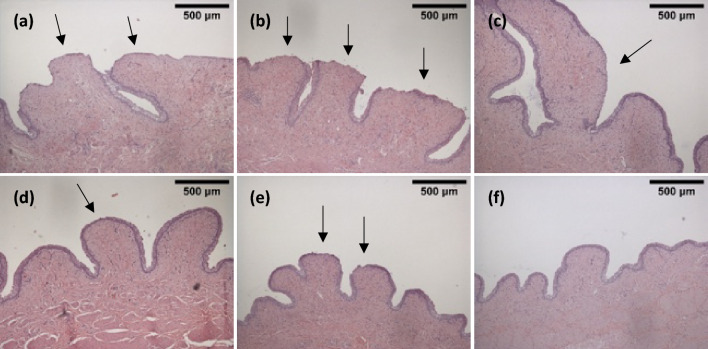
H&E stains of bladder tissue subjected to mucosal suction by the different catheters. (**a**): Brand A, large areas of exfoliation of the urothelium present. (**b**): Brand B, microtrauma similar to brand A large areas of exfoliation of the urothelium present. (**c**): Brand C, areas of exfoliation in the urothelium present. (**d**): MHZC 2, slight thinning of the urothelium. (**e**): MHZC 8, thinning of the urothelium, possible small breach of the urothelium. (**f**): Negative control, no damage to the urothelium. All images at 40 × magnification.

Urothelial microtrauma was present for all CEC brands. The microtrauma caused by these catheters was presented as exfoliation of the urothelium (Fig. 3a–c). As the bladder mucosal suction occurred, the epithelial cells that line the bladder were damaged and dislodged from the basal lamina. The underlying submucosa was exposed by all CECs. The same level of microtrauma was observed for all CECs, as they all induced severe epithelial microtrauma at the areas where the mucosal suction had occurred. MHZCs presented less evident microtrauma than CECs, ranging from slight thinning of the urothelium (Fig. 3d) to small breaches of the urothelium (Fig. 3e). Accordingly, microtrauma induced by MHZCs was substantially less extensive than by CECs. No urothelial changes were seen in any of the control samples (Fig. 3f).

## Discussion

IC is a widely used bladder management approach. While seemingly simple and straight forward, catheterization encompasses a complex interplay of physical phenomena, which may lead to unwanted side effects, such as suctioning of the bladder mucosa into the catheter lumen. Mucosal suction is driven by interactions between different sources of pressure changes inside and outside the catheter, namely the hydrodynamic and hydrostatic pressures. The hydrodynamic pressure is caused by the energy retained in the moving urine column inside the lumen, while the hydrostatic pressure is caused by the intravesical pressure, which is a combination of the intra-abdominal pressure and the detrusor pressure. The hydrostatic pressure is dependent on the body’s position meaning it varies if the patient is standing, sitting, or lying down^23,24^. Sullivan et al. found the corresponding initial resting intravesical pressures to be between 0 and 50 cmH_2_O depending on the individual’s posture^24^. During bladder draining with a catheter, the tissue may come in close contact with the eyelets of the device. When the tissue approaches the eyelet, the flow velocity of the fluid draining into the eyelet is accelerated as the eyelet is closed. Eventually, the tissue is sucked into the catheter eyelet, occluding it and subsequently causing a flow-stop. Following the mucosal suction, the hydrostatic pressure is sufficient to keep the eyelet occluded, even during repositioning^12^. The mucosal suction was detected by Glahn et al. as a hydrodynamically generated negative pressure wave^16^. The blockage of the eyelets by mucosal suction of the tissue with indwelling or suprapubic catheters has been shown to have a harmful effect on porcine bladders and in human clinical trials, resulting in urothelial trauma, mucosal lesions, and edema^17,25^, but the phenomenon is poorly described in IC.

Our study demonstrated the presence of mucosal suction during bladder drainage with CECs and examined the pressure dynamics during mucosal suction in an ex vivo model. The tissue clogged the CEC eyelets and was sucked into the catheter lumen, occluding it. The pressure dynamics recorded during mucosal suction were large negative pressure peaks observed for all 3 brands of CECs, with no significant differences among them. By contrast, as suggested in recently published literature^20^, when using a MHZC instead of a CEC, the pressure dynamics are significantly different, with average negative pressure peaks 4 times less intense than with CECs (approximately -100 to -200 mbar, compared to -800 mbar, p < 0.0001).

In addition to catheter occlusion that may lead to flow-stop during drainage and subsequent insufficient bladder emptying^12,21,22^, a potential consequence of mucosal suction is tissue microtrauma. Earlier research has indicated that mucosal suction occurring inside Foley catheters may indeed result in trauma to the tissue, with mucosal lesions documented with cystoscopy^26^ and higher red blood cell count and hemoglobin in the urine of indwelling non-ventilated catheter users^27^. Furthermore, thinning and exfoliation of the urothelium has also been documented in some pig bladders after mucosal suction through an indwelling catheter^17^, while in dogs the findings included polyploid mucosal marks on the side walls and, in some cases, erythrocytes were found in the urine^15^.

The data discussed here represents the first study to evaluate the impact of mucosal suction on the bladder in IC. The investigations were conducted on a macroscopic level, through naked-eye observation, as well as on a microscopic level, using detailed histology samples. The observations showed that mucosal suction through the eyelets of all 3 brands of CECs assessed in the current study resulted in highly visible macroscopic marks (Fig. 1e), similar to the polypoid mucosal marks reported for indwelling catheters^15^. By contrast, when using the newly developed MHZC the differences were striking, with barely visible macroscopic markings. The micro-holes of the MHZC are cut by thermal laser processes, which eliminate sharp edges. While we acknowledge that the type of eyelets in CECs may impact the extent of the microtrauma, CECs are generally expected to have punched eyelets with edges subsequently smoothened using different manufacturing approaches, like thermal melting. As such major differences are not expected to appear among different CECs. In addition to the data in this paper, where CECs do not show significant differences in terms of pressure drops and mucosal trauma, an earlier publication^12^ also indicated similar behavior in terms of mucosal suction among 3 different CECs.

In addition to macroscopic examination of the samples, histology investigations were conducted. Mucosal suction through all 3 CECs resulted in large areas of exfoliation in the urothelium (Fig. 3abc), similar to results reported in the literature for indwelling catheters^17,26^. At the opposite end, histology results for samples tested with the MHZCs generally exhibited just a slight thinning of the urothelium (Fig. 3de), with a much closer resemblance to the negative control. These findings showed that when using the MHZCs the bladders largely remained intact, preserving the natural biological barrier against potential pathogens and harmful substances^28^. The microscopic findings confirm the macroscopic observations of far less damage resulting from mucosal suction through the micro-holes compared to the eyelets.

It is evident that different catheter eyelet geometries affect the level of microtrauma in the bladder. However, it is unclear what eyelet characteristic is the main culprit for causing the microtrauma. The large negative pressure peaks seen for the CECs (Fig. 2) may be sufficient to cause dislodgement of the urothelium and subsequent damage to the bladder, as energy from the pressure wave is dissipated in the deformation of the tissue. The endoscopic evaluation of the mucosal suction through the CECs, which showed the suction of a large portion of tissue into the catheter lumen, raises the question of microtrauma caused by the stress and strain to the tissue. Furthermore, as repositioning of CECs may often be needed after flow-stops resulting from tissue suctioning inside the lumen^12^, additional microtrauma to the mucosa may occur through mechanical movement during this repositioning, at the contact points between the tissue and the eyelet’s rim. The MHZC addresses these attributes as the negative pressure peaks are significantly less intense and the ingress of the tissue into the catheter lumen is eliminated. These changes are reflected in the low level of microtrauma (Fig. 3). Avoiding urothelial damage may aid in the mitigation of catheter-induced discomfort and pain that affect the life of patients performing IC.

**Figure 4:**
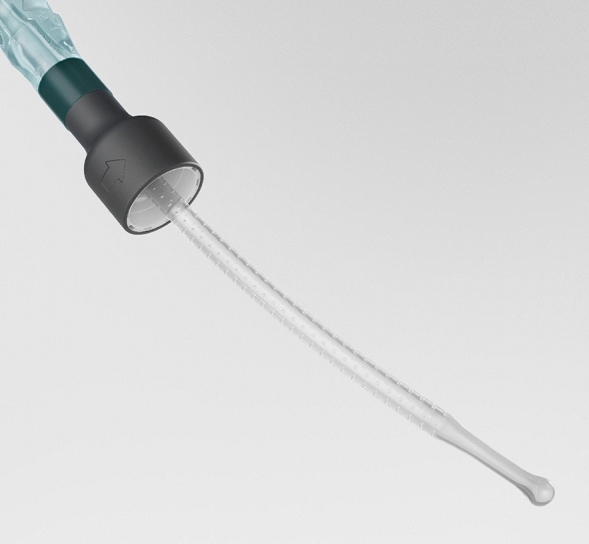
3D render of a proposed catheter with Micro-hole Zone Technology.

When experimenting with the MHZCs, sometimes difficulties in closing all 8 holes of the MHZC 8 simultaneously were observed. This is reflected by the high relative standard deviation of the parameters when compared to the other catheters (Table 1). The morphology of the tissue appeared to be the deciding factor; flow-stops were easier to generate with flat tissue samples. For tissues with varying morphology across the sample, it sometimes proved difficult to occlude all 8 micro-holes simultaneously. This was not experienced with the MHZC 2 prototype. This is consistent with previous results from a study where the full catheter with 80 micro-holes (Fig. 4) was used in a pig lower urinary tract model and where flow-stops were virtually absent with the MHZC^20^. Taken together, the current and previously published results indicate that mucosal suction through the micro-holes of this newly designed catheter is very unlikely and in the rare case when it may occur, the negative impact on the tissue, as reflected by microtrauma, is far reduced compared to CECs, while preserving a similar or superior flowrate^12,20^.

Nevertheless, some limitations exist to this study. Firstly, the potential trauma caused by the catheter tip itself was not investigated, as this was beyond the scope of the current work. It has already been documented in literature that insertion and removal of catheters is itself associated with some trauma^14^, and this paper does not claim to solve all issues related to IC. The work presented here was aimed specifically at understanding what caused mucosal suction through the eyelets of CECs, what its consequences are, and how the side effects may be mitigated. Secondly, while some speculation regarding the potential beneficial impact that reducing microtrauma may have on UTI risks can be made, as maintaining an intact biological barrier to pathogens should, in principle, reduce infection, the work herein does not investigate this important aspect. Furthermore, the clinical impact of bladder mucosal suction and subsequent trauma has not been investigated or assessed definitively in this study or elsewhere, leaving a big knowledge gap to be filled. Thirdly, while using the entire MHZC was not possible due to experimental design, this limitation is, to some extent, diluted by the ex vivo and clinical data already published on the full MHZC^20–22^. Lastly, while this is beyond the scope of this work and prior work indicates that, for the MHZC, little to no mucosal suction occurs regardless of the hydrostatic pressure^20^, for the CECs it may be interesting to assess if there are any differences in the extent of the microtrauma at different pressures.

The results from the present study indicate that a micro-hole zone configuration of an intermittent catheter, with many micro-holes replacing the conventional 2 eyelets, would result in negligible urothelial damage, significantly reduce the negative pressure peaks, and lower the risk of mucosal suction. Such a catheter may prove a useful change in IC technology and could increase compliance with the procedure and patient quality of life.

## Methods and materials

### Catheters

**Figure 5:**
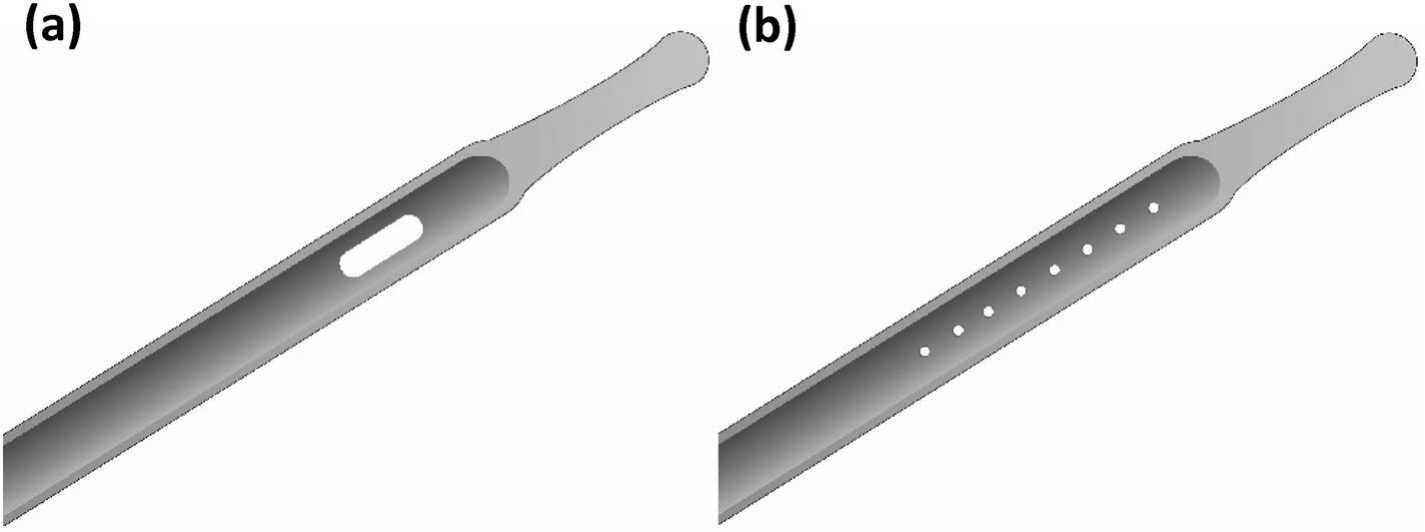
Schematic overview of the catheter types. (**a**): Conventional eyelet catheter (CEC) consisting of typically two large eyelets. (**b**): Micro-Hole Zone Catheter (MHZC) with a drainage zone of many small Micro-Holes. Here depicted with, but not limited to, eight Micro-Holes.

Commercial male urinary Charrière (CH) 12 (also known as French scale or French gauge) CECs from different suppliers were included in this study. These were Coloplast Speedicath Standard, Wellspect LoFric Origo, and Hollister Vapro. These devices are henceforth referred to as brands A, B, and C respectively. While CECs have 2 eyelets, only 1 eyelet was used for the purpose of these experiments, as illustrated in Fig. 5a and described in the following subsections. The single eyelet area of 5.16 – 5.65 mm^2^ was calculated from measurements on scanning electron micrographs, N = 5 per catheter type (TM3000, Hitachi High Tech, Tokyo, Japan). These CECs were compared with 2 prototype MHZC configurations with single micro-hole diameter of 0.4 ± 0.02 mm and holes arranged in rows, with 2 (MHZC 2) or 8 (MHZC 8) micro-holes respectively, spaced 2.1 mm apart, (hereby referred to as experimental MHZC 2 and MHZC 8 (Fig. 5b). The area of a single micro-hole is of 0.127 mm^2^, resulting in a total area of 0.254 mm^2^ for the MHZC 2 and 1.016 mm^2^ for the MHZC 8. Prototypes with 2 and 8 micro-holes were produced to simulate closing of the last 2 micro-holes or a speculative closure of 8 micro-holes (8 was the maximum number of micro-holes that would fit between the connectors of the model while still allowing for tissue to approach the catheter). All catheters with micro-holes were non-commercial intermittent catheters produced by Coloplast A/S solely for investigational purposes. A total of 7 catheters of each type were used for this study.

### Preparation of catheters

**Figure 6:**
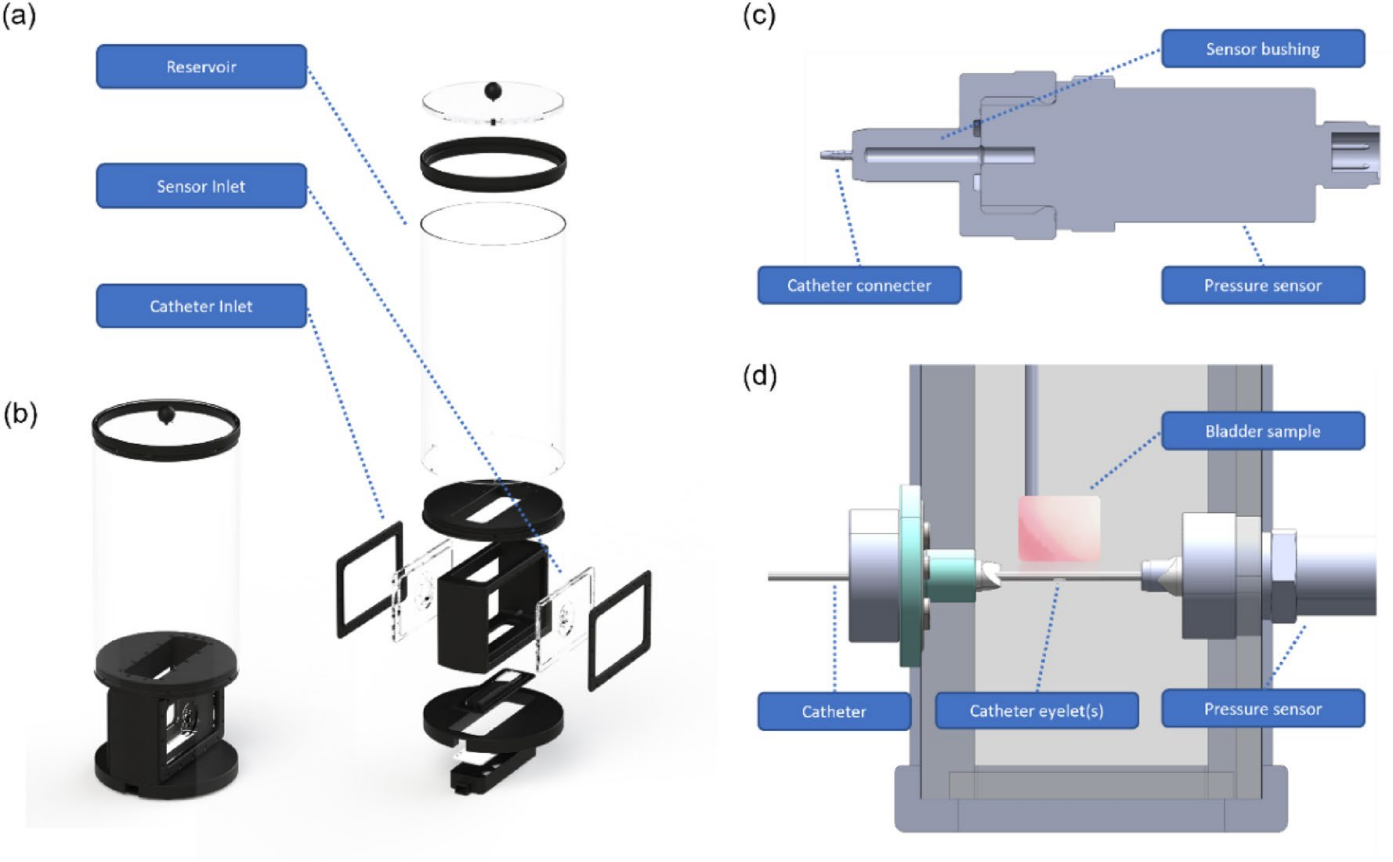
Overview of the experimental setup. (**a**) and (**b**) Renders of the reservoir in exploded and normal view. The model includes a central compartment with two inlets: one inlet for the intermittent catheter and one for the pressure sensor. Situated above the inlet compartment is a reservoir allowing for a static water pressure of up to 50 cmH_2_O (**c**) Cross sectional view of the pressure sensor connected to the sensor bushing which presents a catheter connector of the desired size. (**d**) Cross sectional view of the central compartment with the catheter and sensor inlets. Left is the catheter inlet with a catheter inserted into the inlet and connected to the catheter connecter of the sensor bushing on the opposite side of the central compartment. Also presented is the experimental situation where a bladder sample is slowly moved into close proximity of the catheter eyelet(s) whereafter a mucosal suction occurs.

Since the mucosal suction phenomenon occurs when the last eyelet is closed, the tip and the eyelet closest to the tip were cut off from all tested commercial catheters (brand A, B and C), leaving a single eyelet for all experiments conducted in this study (Fig. 5a). For MHZC 2 and MHZC 8, only the catheter tip was cut off, leaving 1 row of open eyelets. The catheter was then inserted over the catheter connector on the sensor bushing, allowing for the pressure inside the catheter to be measured (Fig. 6b).

### Experimental setup

Taking inspiration from Glahn et al.^16,17^, A model was designed and built to perform the mucosal suction test and measure the pressure fluctuations. The reservoir had 2 inlets on each side (Fig. 6), with an inlet designated for inserting the intermittent catheter and the other one for positioning the pressure sensor (BSP00KM, BALLUFF, Neuhausen auf den Fildern, Germany). The catheter inlet was designed with 2 duckbill valves, creating a water-tight fitting for catheter insertion and removal. A bushing (stainless steel, AISI 304) was designed and produced fitting the pressure sensor for enabling insertion into the reservoir. A hydrostatic pressure of 50 cmH_2_O was applied by filling the reservoir with the appropriate volume of either ≈ 90% degassed (3M™ Liqui-Cel™ MM-1.7 × 8.75 Series Membrane Contactor, 3M, Maplewood, USA) demineralized water for the pressure measurements or isotonic saline (0.9% w/v NaCl, VWR International, Radnor, Pennsylvania, USA—in MilliQ) for the biological evaluations. The volume was maintained by adding new saline (for histology) or degassed deionized water (for pressure readings) throughout testing.

The choice of hydrostatic pressure, which is in the higher range for standing adult humans^24^, was made based on data published earlier and showing similar flowrates through a CEC^12^ and a MHZC with holes of 0.4 mm in diameter^20^ in a porcine lower urinary tract model. While testing of a variety of pressure conditions corresponding to different body postures would be interesting in theory, prior data indicates that for the MHZC there is little if any mucosal suction even at the higher pressures investigated in pig models^20^. With this in mind, the parameters of the model were chosen to represent the worst-case conditions possible, e.g., an abdominal pressure of 50 cmH_2_O mimicking the highest pressures measured for a patient in a standing position^23,24^, ensured closing of the last eyelet of the catheter, and repeated mucosal suctions for each sample as repositioning^12^ the catheter could realistically cause several mucosal suctions. The 3 different brands of commercially available, standard of care CECs were tested against 2 prototypes with 2 and 8 micro-holes respectively, to simulate potential closure of a MHZC. The tests included a visual inspection of mucosal suction in the catheters with an endoscope, pressure recordings during mucosal suction from inside the catheter lumen, and an analysis of microtrauma with histology.

### Preparation of porcine tissue

The ex vivo experiments were performed on fresh bladder specimens of pigs obtained from a local **s**laughterhouse (Glumsø Slaughterhouse ApS, Glumsø, Denmark). The bladders were delivered with the urethra attached. The bladders were emptied of urine and excessive tissue was trimmed away. The urethra was discarded along with the bladder neck and apex. Samples of approximately 2.5 × 4 cm were made by sectioning the bladder tissue. The samples were then attached to cork pieces with needles through the detrusor muscle at the corner of the samples to support the tissue and ease processing for histology. A customized sample holder, comprised of a rod with clamps, was used to submerge and control the bladder sample in the filled reservoir.

### Visual inspection of mucosal suction

The sensor inlet was changed to a different fixture allowing for the insertion of an endoscope into the catheter lumen through the hole created in the cut tip, ensuring normal flow conditions. A pressure of 50 cmH_2_O was applied by filling the reservoir with tap water, and the visual inspection was performed inserting a rigid endoscope (MEDIT Inc., Winnipeg, Manitoba, Canada) into the catheter. The catheter and the endoscope were inserted in the model in the 2 separate inlets. The endoscope was then moved into the lumen of the catheter through the tip. An endoscope diameter of 2.7 mm ensured complete closure of the catheter. The bladder specimens were prepared, and a single mucosal suction was performed. The endoscope was placed approximately 5 mm from the eyelet or micro-hole and the phenomenon of mucosal suction was recorded with the endoscope from inside of the catheter lumen. Images of the tissue were captured (EOS 1300D, Canon Inc., Tokyo, Japan) and descriptive images were taken immediately after occurrence of 5 repeated mucosal suctions with a single catheter of each type in a single bladder.

### Pressure measurements during mucosal suction

**Figure 7:**
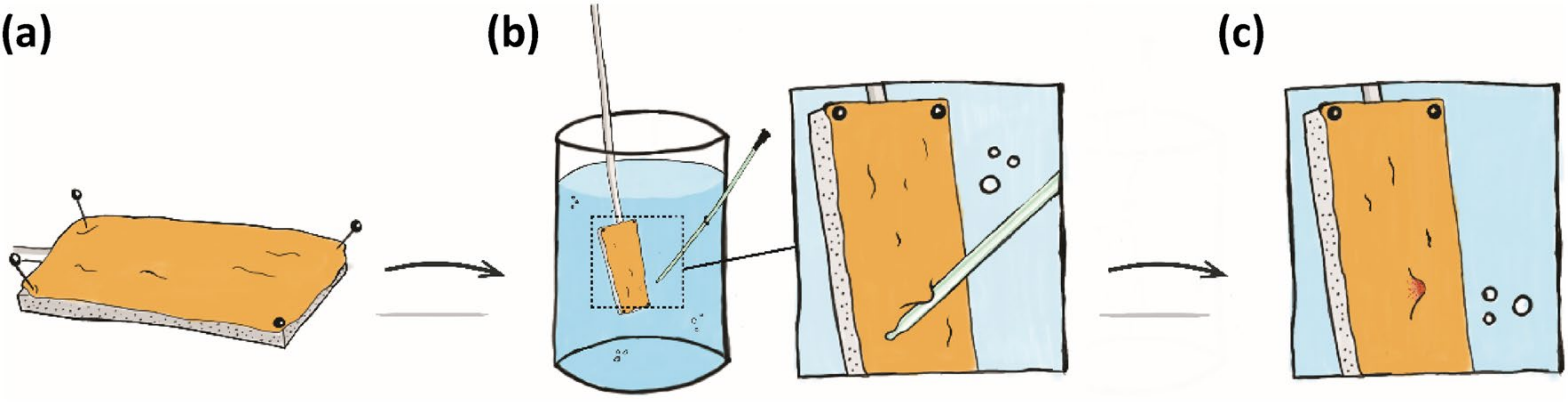
Overview of the workflow for creating mucosal suctions on tissue. (**a**): Porcine bladder tissue is attached to a piece of cork with needles. (**b**): The tissue is lowered into the reservoir and moved close to the eyelet(s) of a catheter draining the fluid from the reservoir. (**c**): A distinct suction mark is left on the tissue; this is called a mucosal suction.

For each type 7 catheters (35 in total) were tested in 3 different porcine bladders for the pressure readings. The submerged porcine bladder sample (Fig. 7a) was slowly advanced towards the eyelet of the given catheter and when the tissue came in close proximity to the eyelet, the bladder mucosa was sucked into the eyelet (Fig. 7b). After a few seconds (< 5 s), the bladder tissue was detached from the eyelet by moving the tissue away from the eyelet to release the suction, leaving a suction mark on the tissue (Fig. 7c). This re-established the flow through the catheter. The bladder mucosa suction process was repeated 10 times for each tissue sample, with the same area of the tissue samples being deployed for suction, resulting in multiple pressure profiles for each sample. Each of the 7 catheters of each type was tested in 3 different bladders for a total of 21 tissue samples per catheter type. Data was logged (USB NI 6351, National Instruments, Austin, Texas, US) and collected in NI DAQExpress (National Instruments, Austin, Texas, US).

The pressure traces were processed in Python (Python Software Foundation, www.python.org), all the traces were zeroed to the pressure recorded during undisturbed flow through the catheter, and peaks were found. Statistical analysis comprised of Brown-Forsythe and Welch ANOVA tests with a Dunnet’s T3 multiple comparisons t-test, summary statistics, and final plots were made with GraphPad Prism 9 (Dotmatics, Boston, Massachusetts, USA).

### Trauma assessment and histology

A single representative catheter of each type was chosen based on the median peak pressure for that catheter type found in the pressure recordings. Each of the catheter types was tested in 3 different bladders for a total of 21 samples including a negative control sample from each bladder. These samples were used for histological analysis. Mucosal suctions were performed as described in the previous subsection, but only 5 tests were run on each bladder sample. The 3 CECs were tested on tissue pieces from the same 3 bladders, while the MHZCs were tested in 3 different bladders due to limitations arising from bladder sizes. All the bladders were allocated randomly and were from both male and female pigs. The 21 tissue samples were subsequently subjected to histological analysis to evaluate if the mucosal suction had induced any mechanical damage to the bladder tissue. The location of the mucosal suction was marked on the cork base with a permanent marker immediately after sample retrieval from the model. The tissue was subsequently fixed in 4% formaldehyde pH 6.9 (Sigma-Aldrich, St. louis, Missouri, USA) with the samples still attached to the cork. The samples were sent to IN-LAB (IN-LAB ApS, Virum, Denmark) pathology lab where the location of the mucosal suction was marked on the edges of the tissue according to the markings on the cork. The tissue sample was then split into 2 parts. A part had the suction mark, while the other did not and served as the sample specific control to assess for the quality of the individual sample and slice. Both parts of the sample were paraffin embedded together. The samples were trimmed until the colored marks on the side of the tissue, denoting the location of the mucosal suction, became visible. Hereafter, sections of 8 µm thickness were collected on glass slides and H&E stained. The slides were examined with light microscopy (BX60, Olympus, Tokyo, Japan). The analysis was non-blinded. Scalebar was added in Fiji^29^.

## Data Availability

Raw data are available upon request to the corresponding author.
